# Theoretical evaluation of mental health first aid using the behavioural change wheel (BCW)

**DOI:** 10.1080/21642850.2026.2623324

**Published:** 2026-01-30

**Authors:** Opeyemi Atanda, Kerry Wood, Patrick Callaghan, Paula Reavey, Eleni Vangeli

**Affiliations:** a School of Allied Health and Life Sciences, London South Bank University, London, UK

**Keywords:** Help-seeking behaviour, behavioural change wheel, theoretical domain framework, behavioural change techniques, mental health first aid

## Abstract

**Background:**

Mental health first aid (MHFA) has gained popularity over the years. This study retrospectively maps the MHFA intervention to characterise its content using the Behavioural Change Wheel to identify the active ingredients and mechanisms of action.

**Methods:**

Three data sources formed the data for the current study. Namely, the MHFA training manual, a roleplay video demonstrating how to implement the intervention, and interviews with six participants of whom five were MHFAers and five were MHFA recipients, on their experiences of the MHFA intervention. The interview data source formed part of the EMPOWER trial. The study presented here utilised a two-step approach: a) The MHFA intervention was described using the Template for Intervention Description and Replication (TIDieR) framework, and b) the content was analysed to identify the behavioural change techniques (BCTs) using the behavioural change techniques taxonomy version 1 and intervention functions using the behavioural change wheel. The mechanisms of action were specified using the capability, opportunity, motivation model of behaviour and theoretical domains framework.

**Results:**

Twelve BCTs and four intervention functions were identified across the three data sources. Only social support BCTs and the intervention functions of enablement and persuasion were consistently identified across the three data sources in the MHFA. The most frequent mechanisms of action were reflective motivation (particularly ‘beliefs about capabilities’ and ‘goals’) and social opportunity (particularly ‘social influences’).

**Conclusions:**

The MHFA intervention incorporates BCTs to increase reflective motivation and social opportunity to seek help for mental health concerns. However, psychological capability and physical opportunity need to be addressed to enhance help-seeking behaviours in those experiencing mental health difficulties. Future research should evaluate the impact of integrating additional BCTs and intervention functions into the MHFA programme to determine the most effective combination for promoting help-seeking behaviour and improving mental health outcomes.

## Introduction

Mental health remains a global concern, with one in eight people living globally with mental health difficulty (World Health Organization, 2022). Seven percent (7%) of all health-related issues in the UK are mental health difficulties (King's Fund, 2024). The prevalence of common mental health difficulties (typically high-prevalence conditions such as depression, anxiety disorders, panic disorder, phobias, obsessive–compulsive disorder (OCD), and posttraumatic stress disorder (PTSD)) is approximately 16.9% among adults in England (Public Health England, 2021). Over the years, improving health literacy has emerged as a vital public health drive to increase positive health outcomes (Kutcher et al., 2016). Mental health literacy (MHL) emerged from health literacy and was developed by Jorm and colleagues (Jorm et al., 1997). The concept includes knowledge that helps with early recognition of mental health issues, self-help strategies, and first aid skills to support anyone experiencing mental health difficulties. Other components involve understanding self-treatment options and available professional support, as well as efforts to shift attitudes to promote recognition and appropriate help-seeking. The challenge of recognising causes and risk factors related to mental health issues is one of the many barriers to seeking help (Gulliver et al., 2010).

Mental health first aid (MHFA) enhances mental health literacy, allowing non-clinical workers to receive minimal training for delivery in workplaces, schools, and communities, including social networks of family and friends. As with physical first aid (Eisenburger & Safar, 1999), MHFA includes an action plan to offer initial assistance to an individual experiencing a mental health difficulty or crisis. The five steps are approaching the person and assessing/assisting with any crisis, listening non-judgementally; giving support and information, encouraging the person to obtain appropriate professional help, and encouraging other support (ALGEE). Importantly, the ALGEE action plan, the structured five-step model taught in the MHFA, was not developed in isolation but was grounded in a substantial body of Delphi expert consensus studies. These studies systematically engaged clinicians, consumers, and carers to identify the most appropriate public responses to a wide range of mental health problems and crises. Items endorsed by at least 80% of the expert panels were incorporated into guidelines, which were subsequently synthesised into the ALGEE framework and embedded within MHFA curricula (Jorm & Ross, 2018). This evidence-based process ensures that ALGEE aligns with international best-practice first aid for mental health. However, limited evidence confirms if following these guidelines improves outcomes. The MHFA plan helps trained people encourage those with mental health struggles to seek help early for appropriate support.

MHFA has gained popularity and has become the focus of government funding (Atanda et al., 2020b). In 2017, Public Health England (PHE) announced £15 million towards training up to one million people in MHFA skills. Additionally, £5 million has been invested in teachers' training to address the mental health issues affecting primary school pupils (Department of Health and Social Care, England PH, 2017). MHFA has also been implemented to engage with UK employers concerning mental health awareness (Harvey et al., 2014). Despite the growing popularity, evidence is less convincing in demonstrating that MHFA interventions improve access to professional services for those identified or diagnosed with mental health difficulties (Jorm et al., 2007). Despite the increased number of evaluation studies within the workplace context, little has been done to investigate the impact of MHFA on colleagues who engaged with MHFAers (recipients) (Kitchener & Jorm, 2006; Wong et al., 2019).

Recently, an evidence synthesis by Atanda et al. (2020b) examined the effectiveness of MHFA across various outcomes, the contexts in which it has been applied, and its mechanisms of action. It found that the ALGEE approach, the guiding principle overseeing the MHFA intervention, lacks theoretical or conceptual clarity on how it achieves its outcomes. The synthesis also pointed out a lack of evidence regarding its effectiveness for intervention recipients, mainly because assessing these outcomes is challenging. It highlights several barriers and facilitators to implementing MHFA. A key obstacle is persistent stigma or negative attitudes toward mental health in certain settings, which can prevent individuals from seeking help or providing support. On the other hand, successful MHFA implementation is often supported by strong leadership commitment, an organisational culture that encourages openness and positive attitudes about mental health, and clear roles and responsibilities for MHFA volunteers (Carpini et al., 2024). Recently, the Cochrane review by Richardson et al. (2023) analysed data from 21 RCTs involving over 22,600 participants. It assessed outcomes like mental health, use of mental health services, and adverse events in communities that had trained MHFAers. The review found that the evidence is of very low certainty due to the lack of good-quality evidence, so no definitive conclusions can be made about the benefits to its recipients.

Despite the emphasis on using theory to develop and evaluate interventions (Moore et al., 2015), several studies evaluating behavioural interventions do not present a theoretical model that underpins them (Kleinman & Dougherty, 2013; Marcus et al., 2006). Consequently, Michie and colleagues (Michie et al., 2005) developed the behavioural change wheel (BCW) to support theory-based development of behaviour change interventions.

The BCW, developed from a systematic review of 19 behavioural change frameworks (Michie et al., 2011), provides a systematic method for conducting a behavioural diagnosis of what needs to be changed to enable the desired behaviour and for developing relevant interventions and supportive policies to facilitate it. It comprises three layers: the COM-B (‘capability’, ‘opportunity’, ‘motivation’, and ‘behaviour’) model forms the wheel's hub, in which behaviour is part of an interacting system involving three essential components. The theoretical domains framework (TDF) allows a more comprehensive behavioural analysis that maps onto the COM-B model and consists of 14 theoretical domains of explanatory constructs, identified from a synthesis of 33 behaviour change theories developed using a consensus approach (Cane et al., 2012). Surrounding the COM-B model is a layer of nine intervention functions that can be used to create behaviour change. These intervention functions are linked to behaviour change techniques (BCTs), which are the smallest active components designed to change behaviour (e.g. strategies such as action planning or self-monitoring). The behaviour change technique taxonomy (BCTTv1) provides a standardised framework for reporting the ‘active ingredients’ of an intervention (Michie et al., 2013). The outer layer of the wheel identifies seven policy categories that can support the delivery of the intervention functions.

The BCW has been adopted in the development of behavioural change interventions, including non-clinical interventions, like changing sitting time amongst office staff (Ojo et al., 2019), increasing the intention to post anti-littering messages on social media (Kolodko et al., 2021), increasing physical activities (Truelove et al., 2020), and promoting physical activities of young people at risk of psychosis (Carney et al., 2016). It has also been utilised in various health-related activities, such as addressing prescribing errors and promoting proper hand hygiene (Steinmo et al., 2015).

In addition, the BCW has increasingly been applied to analyse an intervention retrospectively to unpack its theoretical content and mechanisms of action to change a target behaviour. In the case of MHFA, the target behaviour is seeking professional help following an encounter with an MHFAer. Retrospective analysis is limited to the intervention content available in existing training materials and experiential accounts and can therefore only infer the implicit mechanisms of action from the identified BCTs. Nevertheless, in the absence of a pre-existing theoretical framework this retrospective analysis provides a standardised approach to identify how the intervention's activities are expected to bring about behaviour change. The TDF 14 domains (e.g. knowledge, skills, social influences, beliefs about capabilities, etc.), provide a more fine-grained understanding of the psychological and contextual factors (barriers and enablers) that influence behaviour than the COM-B. By mapping components of an intervention onto the COM-B and TDF domains, one can identify which capabilities (physical or psychological skills, knowledge), opportunities (social and environmental supports), and motivations (reflective and automatic drivers) the intervention targets.

The current study draws on approaches taken in previous studies (McHugh et al., 2018; Powell & Thomas, 2022; Steinmo et al., 2015) that have utilised the tools above to retrospectively characterise interventions using multiple data sources. The current study aims to a) use the BCW to report the theoretical content of the MHFA intervention, i.e. the ALGEE approach in practice and b) characterise its potential theoretical mechanisms of action (i.e. how and why it might lead to changes in behaviour and outcomes). This study formed part of the EMPOWER trial (Atanda et al., 2020a), which evaluated the effectiveness of MHFA on workers in several UK organisations. The target behaviour is recipient's help-seeking, which occurs after an encounter with an MHFAer. This was also the primary outcome in the EMPOWER trial.

## Methods

This study used a retrospective mapping exercise of elements of the BCW to the MHFA intervention aimed at promoting recipients' help-seeking behaviour. The mapping exercise took a two-step approach to: 1) deconstruct the MHFA intervention using the TIDieR framework (Hoffmann et al., 2014) and 2) identify BCTs, intervention functions, and potential theoretical mechanisms of action. The second step involved a three-stage process to map the intervention to the BCW and TDF.

### Sources of data

Data on intervention content were gathered from three sources, selected in consultation with MHFA England, who were the original funders of the EMPOWER Trial. The MHFA training manual and the role-play video, both used in the training of MHFAers, were provided as key resources.

1. MHFA training manual – This manual accompanies the MHFA training. The training manual reviewed is the MHFA England manual, which is based on the third edition Australian MHFA manual (Kitchener et al., 2013). The manual includes a general introduction to the ALGEE approach, four sessions to illustrate the ALGEE approach applied to different mental health conditions, and a directory of mental health support sources. The session on applying the ALGEE approach in the context of a recipient experiencing (or showing behavioural symptoms of) *psychosis* was selected as data source 1 (Session 4, Page 188–219). This session was selected to match a role-play training video ‘good practice’ example used in the MHFA training programme to demonstrate the ALGEE approach and facilitate cross-source comparison.
2. MHFA Role-play Video – A role-play training video on psychosis was obtained from MHFA England. This included both a good and a bad example of interventions with an individual experiencing a psychotic episode, with the good example demonstrating MHFA in action. In the good example, a trained MHFAer utilised the ALGEE action plan to support an individual in a psychotic episode.
3. Interviews – Of the 24 participants interviewed as part of the EMPOWER trial, five were MHFA recipients and 10 were MHFAers. The current study conducted a secondary analysis of a subset of these interviews that met the inclusion criterion of the participant being either an MHFAer who reported supporting a colleague in the capacity of MHFAer since their training 6 months earlier (*n* = 5) and/or an individual who had received support from an MHFAer in the last 6 months (*n* = 5). Four MHFA recipients reported also supporting a colleague as an MHFAer and so narrated their account from their experiences as both MHFAer and recipient. A total of six interviews were examined in the current study (Atanda et al., 2020a). The interviews were conducted six months post-training. The section of the interview where participants recounted their experiences of administering or receiving a MHFA support was examined in the current study. Recipients were asked to describe ‘the kind of support they received and how it affected their feelings and mental health’. They were prompted to describe specific qualities they identified during their encounter with the MHFAer. MHFAers were asked to describe ‘the instances where they applied their MHFA skills post-training’. They were prompted to provide a detailed example of their encounter, explicitly identifying the application of the ALGEE approach. Those who were both recipients and MHFAers were asked to describe instances when they received MHFA support and when they supported someone. The analysis sought to identify active ingredients in 1) trainees' descriptions of how they have supported any individual following the training and 2) recipients' perceptions of the support received and their interaction(s) with the MHFA.

### Data analysis

Step 1. The MHFA intervention was deconstructed according to the TIDieR framework (Hoffmann et al., 2014). The MHFA intervention included the rationale for the intervention, materials used to deliver the intervention, procedures involved when a MHFAer administers MHFA, and the frequency of contact with a recipient. Data from all three sources were used.

Step 2 (Stages 1, 2 and 3). This step consists of three stages to map the intervention content from each data source according to the guidance and tools provided in the BCW framework (Michie et al., 2014). Help-seeking behaviour among recipients was identified as the target behaviour, consistent with the EMPOWER trial primary outcome, following consultation with the funders, MHFA England. A consensus approach was adopted in each of the three stages to identify BCTs, intervention functions, and mechanisms of action, drawing on the training manual and video. That is, two researchers (OA and KVW) independently conducted the analysis. Following the independent reviews, the researchers met at each stage to discuss the findings, resolve discrepancies, and reach a consensus with a *third* researcher (EV), who has specific expertise in applying the BCW framework. For the interview data, the BCTs, intervention functions and mechanisms of action were identified by OA and discussed with EV to ensure appropriate grounding in the data.

*Step 2: stage 1.* The intervention content was analysed to identify observable active ingredients (BCTs) in MHFA that promote help-seeking behaviour, using the BCTTv1 (Carney et al., 2016).

*Step 2: stage 2.* The BCTs identified in stage 1 were then linked to the intervention functions (broad categories of actions that can be taken to influence behaviour) to illustrate how MHFA aims to achieve its intended outcome of promoting help-seeking behaviour outcome.

*Step 2: stage 3.* The BCTs with the corresponding intervention function were then mapped to the TDF COM-B model to identify mechanisms of action.

## Results

### Step 1

Table 1 summarises the overall content of the MHFA intervention utilising the Template for intervention description and replication – TIDieR framework (Hoffmann et al., 2014). Recipients of the intervention are individuals struggling with mental health difficulties (depression, suicidal crisis, anxiety disorders, eating disorders, personality disorders, self-harm and psychosis were explicitly discussed in training). It outlines the brief name (MHFA), the rationale (to improve mental health literacy and equip individuals to recognise, respond to, and support others experiencing mental health difficulties), and the materials provided (two-day interactive training, manual, workbook, and role-play video). The procedures are described through the five ALGEE steps (approach/assess, listen, give support, encourage professional help, encourage other supports), which guide MHFAers in responding to crises. The delivery context is detailed, highlighting that MHFA is applied in person when an individual shows signs of a mental health crisis in the workplace, with no prescribed frequency of contact.

**Table 1.** Summary of the overall content of the MHFA intervention mapped to the TIDieR framework.

TIDieR checklist	Description
Brief name that describes the intervention	MHFA intervention
Rationale (why)	The intervention was created to improve mental health literacy. It focuses on recognising mental health issues, seeking reliable information, understanding potential causes and risk factors, and learning about self-treatment and professional help options to intervene and support individuals experiencing mental health difficulties.
Materials	Individuals are trained via a 2-day face-to-face interactive training workshop; they are given a training manual and workbook, which includes detailed steps on how to support individuals experiencing mental health difficulties. Also, participants are shown a role-playing video during the training on administering the intervention.
What procedure	MHFAiders are expected to use five-step action points to support anyone experiencing a mental health crisis. It is called the ALGEE steps, which are. A – Approach the person, assess, and assist with any crisis. L – Listen non-judgmentally. G – Give support and information. E – Encourage the person to get appropriate professional help. E – Encourage other supports.
When and how often	Intervention is delivered in person when a person appears to be experiencing a mental health crisis. The intervention occurs whenever an individual at the workplace presents with a mental health difficulty crisis, which may be identified via observation of the individual's behaviour or raised directly with MHFAider via self-referral. The training materials do not specify the frequency of contact once the individual has received MHFA.

Note: A reduced TIDieR checklist was used to capture the essential features of MHFA (name, rationale, materials, procedures, delivery) relevant to mapping intervention content to BCTs, intervention functions, and mechanisms of action.

### Step 2 (stages 1 and 2)

Twelve BCTs were identified across the three data sources and are presented in Table 2. Only one BCT was shared amongst all three data sources: social support (practical). Two BCTs, ‘action planning and problem solving’ were present in two data sources (MHFA role-play video and Interview transcripts). Two intervention functions were identified across the three data sources (persuasion and enablement), with a further two (education and coercion) identified in one source.

**Table 2.** BCTs and intervention functions observed in each data source.

BCTs from MHFA role play video	BCTs from training manual	BCTs from interview transcripts of support
Goal setting (behaviour) Problem-solving Action planning Social support (practical) Social comparison Future punishment Framing/reframing	Social support (unspecified) Social support (practical) Social Support (Emotional) Information about health consequences Focus on past success	Social Support (Emotional) Social Support (Practical) Action planning Problem-solving Verbal persuasion about capabilities

Intervention function (MHFA role play video)	Intervention function (training manual)	Intervention function (interview transcripts)
Enablement Persuasion Coercion	Persuasion Education Enablement	Enablement Persuasion

### Step 2 (Stage 3)

Tables 3–5 show the BCTs and intervention functions, together with expected mechanisms of action via links to the broad components of Capability, Opportunity and Motivation, and then more specifically to the domains of the TDF. Most of the intervention content related to reflective motivation (*n* = 10) and social opportunity (*n* = 9). One BCT was linked to Psychological ‘Capability’ and, similarly, another linked to Automatic ‘Motivation’. No intervention content was linked to the physical components of capability and opportunity. The intervention content was linked to 6 of the 14 TDF domains. The domain of ‘social influence’ was the most frequent mechanism of action (*n* = 12), targeted via the ‘enablement’ function using a specified or unspecified social support BCT in all but one instance, with the other operating via the function of ‘persuasion’ with the BCT of social comparison. The next most frequent TDF domains were ‘goals’ (*n* = 6), which were targeted via the function of ‘enablement’ using a variety of BCTs e.g. goal setting (behaviour), problem-solving, action planning, social support (practical), and ‘beliefs about capability’ (*n* = 3) primarily targeted via the function of ‘persuasion’ via the ‘verbal persuasion of capability’ and ‘focus on past success’ BCTs. The other TDF domains targeted were ‘knowledge’ (*n* = 1) ‘reinforcement’ (*n* = 1) and ‘beliefs about consequences’ (*n* = 1).

**Table 3.** MHFA role-play video: characterising intervention content and mechanisms of action using the BCT taxonomy (v1); behaviour change wheel; capability, motivation, behaviour model; and TDF.

MHFA role-play video				
	Intervention content		Mechanisms of action	
	BCTs	Intervention functions	COM-B	TDF
‘Which other options do you want to go with?’ ‘I guess the crisis team’. (10:11)	Goal setting (behaviour)	Enablement	Motivation (reflective)	Goals
Offering different help-seeking strategies and prompting the identification of barriers and options to select (10:13).	Problem-solving	Enablement	Motivation (reflective)	Goals
‘Would you like me to call them here, and you can listen to what I say?’ ‘Yes’ (10.21).	Action planning	Enablement	Motivation (reflective)	Goals
Connecting him to the crisis team (08:18)	Social support (practical)	Enablement	Opportunity (social)	Social Influences
Helper asks what he would do if it were the other way around (referring to Peter helping her) would not you want to help me? (9.31)	Social comparison	Persuasion	Opportunity (social)	Social Influences
Threat of calling the police if you do not seek help action plan (10.00)	Future punishment	Coercion	Motivation (automatic)	Reinforcement
The neighbour was reframing his belief about the intentions of the crisis team if they were called to come to his aid. (7:54–08:28)	Framing/reframing	Persuasion	Motivation (reflective)	Beliefs about consequences

**Table 4.** MHFA training manual: characterising intervention content and mechanisms of action using the BCT taxonomy (v1); behaviour change wheel; capability, motivation, behaviour model; and TDF.

MHFA training manual				
	Intervention content		Mechanisms of action	
	BCTs	Intervention functions	COM-B	TDF
Try to find out whether the person has a supportive network and, if they do, encourage them to get support from these people. Family and friends will be better able to support their loved ones, such as local carer's groups or online peer support groups (Pg 214).	Social support (unspecified)	Enablement	Opportunity (social)	Social influences
Try to find out if the person has anyone they trust (e.g. close friends, family) and try to get them to help (Pg 205). If the person decides to seek professional help, you should make sure they are supported emotionally and practically in accessing services (Pg 207).	Social support (unspecified)	Enablement.	Opportunity (social)	Social Influences
Offer the person choices of how you can help them (Pg 207). If the person has an advance directive or mental health crisis card, you should follow those instructions. (These are pre-written plans which allow a person with a diagnosed mental health issue to communicate their preference about future treatment and care in advance).	Social support (practical)	Enablement	Opportunity (social)	Social influences
You should be prepared to call for help from emergency services (Pg 205).	Social support (practical)	Enablement	Opportunity (Social)	Social Influences
If the person decides to seek professional help, you should make sure they are supported emotionally and practically in accessing services (Pg 207).	Social support (emotional)	Enablement	Opportunity (Social)	Social Influences
Reassure them that you are there to help and support them (Pg 207).	Social support (emotional)	Enablement	Opportunity (Social)	Social Influences
Convey a message of hope by assuring them that help is available and that things can get better (pg 207).	Social support (emotional)	Enablement	Opportunity (Social)	Social Influences
Offer information – give them resources that are accurate and appropriate to their situation (Pg 207).	Information about health consequences	Education	Capability (Psychological)	Knowledge
You could ask them if they have felt this way before and, if so, what they have done in the past has been helpful (Pg 207).	Focus on past success	Enablement	Motivation (Reflective)	Beliefs about capabilities

**Table 5.** Interview transcripts: characterising intervention content and mechanisms of action using the BCT taxonomy (v1); behaviour change wheel; capability, motivation, behaviour model; and TDF.

Interview transcripts				
	Intervention content		Mechanisms of action	
	BCTs	Intervention functions	COM-B	TDF
‘And then it was making sure that you know, I am encouraging them to reach out; it was not something too serious, but it was, you know, just making sure that they know, what was going on there’.	Verbal persuasion about capability	Persuasion	Motivation (reflective)	Beliefs about capabilities
‘And seeing how they felt about certain things, including speaking to their manager, including, you know, as I said, you know, what, you can do it on your own or, you know, anyway that you want to do it, or we don't involve them at all, that's fine. Making sure that they knew. You know, if they wanted a doctor's appointment, they could go make one; I can go with them if they want to signpost some of the, like the talking, as well’.	Verbal persuasion about capability	Persuasion	Motivation (reflective)	Beliefs about capabilities
‘So, it was not really a case of having to offer any particularly practical support. But we did sort of talk it through, you know, it was an opportunity for her to discuss some of her problems’.	Social support (emotional)	Enablement	Opportunity (social)	Social Influences
‘I provided them with a number of resources afterwards to say, let these or I talk it through in the meeting. And then followed up with the resources afterwards to what they could; I guess the main resources that I provided were internal resources’.	Social support (practical)	Enablement	Opportunity (social)	Social Influences
‘So what I have done is pointed them towards some resources, open the door for a chat, if they want to chat, sometimes most of the time, they do not know, they just want to know where the resources are, because they ca not find them on our internal sites. And when people are struggling, they just want answers quickly, sometimes, you know, they are brave enough to reach out. So it is basically just giving them point them towards the resources, saying I'm there for a chat if they need it’.	Social support (practical)	Enablement	Opportunity (social)	Social Influences
‘The key focus of like the conversation we had, it was a really good opportunity to talk trying to establish if there was anything within the working environment, which was causing issue there and try and offer some practical steps in that area in terms of, you know, working with any of her managers and things like that to balance workload. But in terms of giving advice, it was really on the kind of work front, as I say she was really in touch with her G.P. about it. There was not anything she specifically needed from like external support’.	Problem-solving	Enablement	Motivation (reflective)	Goals
‘So, getting very upset, you know, not really being able to feel that they could come into work. So I had some discussions with them, talked about what they wanted to do, you know, how to calm them down, sort of saying to them, you know, go out for a walk at lunchtime, or, you know, when you get home in the evenings because it was a summer, you know, go and get some fresh air, try and clear your mind, do some breathing, and then maybe just talk through with her about what her options were about what she wanted to do. And I did not tell her what to do’.	Action planning	Enablement	Motivation (reflective)	Goals
‘So, it was more kind of assessing what do you need right now? So, yeah, it was useful to like work through the model and listen to what they had to say, give them support and resources in terms of advice of where maybe helpful for them to look at more information on and then encourage sort of the professional help’.	Problem-solving	Enablement	Motivation (reflective)	Goals

Note: Intervention content is specified using behaviour change techniques (BCTs) as the smallest active ingredients, organised under relevant intervention functions (e.g. education, training, modelling, enablement). Mechanisms of action are mapped onto the COM-B model (capability, opportunity, motivation) and the theoretical domain framework (TDF), which provides more detailed explanatory constructs through which MHFA interventions achieve help-seeking behaviour outcomes (e.g. knowledge, skills, beliefs, social influences). This mapping illustrates the pathways from intervention content to behaviour change.

## Discussion

The current study describes a retrospective process of undertaking a systematic and theory-based approach to identify the active ingredients and mechanisms of action of MHFA to improve the help-seeking behaviours of recipients of the intervention. Twelve BCTs and four intervention functions were identified across the data sources. The study identified five mechanisms of action from the TDF (COM-B component shown in brackets) to stimulate help-seeking behaviour. The most common being ‘social influence’ (social opportunity), which is achieved through the ‘enablement’ and ‘persuasion’ functions, and ‘goals’ (reflective motivation), which is achieved via the ‘enablement’ function. Other mechanisms of action include ‘knowledge’ (psychological capability), ‘environmental context and resources (physical opportunity) and ‘beliefs about capability’ (reflective motivation).

Identifying social support as a primary active ingredient in MHFA for enabling help-seeking behaviours across multiple data sources highlights the importance of interpersonal relationships to facilitate access to mental health support. However, it also raises questions about the types of social support most effective across various contexts. This variation in the types of social support recognised across the different data sources (i.e. emotional, physical, unspecified) prompts us to consider the nuances of social support and how they might impact the effectiveness of mental health interventions aimed at promoting help-seeking behaviour. A previous correlational study that examined the relationship between social support and help-seeking before deliberate self-harm, reported that there was a significant link between the quality of practical and emotional support from existing personal networks and the likelihood of seeking help for mental distress and suicidal thoughts, with those with better quality support more likely to seek help (Wu et al., 2011).

The current study findings indicate that future interventions may benefit from the identification of BCTs that support the implementation of each element of the ALGEE action plan. While there were some BCTs identified that address the LGEE elements of the action plan, there were no BCTs identified that relate to the A (approach) element. The first step (A- approach) is not informed by any BCTs and was mentioned as difficult to implement by all MHFAers. They explained that unless the person is visibly in a crisis, it was unclear how to identify whether a colleague needs support from an MHFAer. This indicates a need to support MHFAers in implementing the approach element of the action plan. The current absence of this may at least in part explain why, in the main trial (EMPOWER) (Atanda et al., 2020a), only six employees reported receiving MFHA support out of 500 employee responders. Although no BCT explicitly addresses the approach element of the MHFA, using BCTs such as ‘restructuring the social environment’ can be beneficial. This can support individuals with concealable identities, like those with mental health difficulties, by providing support even when they are not openly seeking help. The ‘restructuring the social environment’ BCT can support MHFA implementation by normalising mental health conversations and fostering peer support, making it seamless for MHFAers to approach and engage with colleagues. For example, via integration of designated times for informal well-being check-ins or embedding MHFAers role into team routines. This may reduce stigma and create opportunities for proactive, supportive interactions. Such changes create a culture where staff feel safe approaching and using MHFA resources effectively.

The theoretical domains that direct behaviour change via the component of capability are ‘knowledge’, ‘skills’, ‘memory’, ‘attention and decision processes’ and ‘behavioural regulation’. However, except for the presentation of ‘knowledge’ in the manual to guide the MHFAer to offer an individual with mental health difficulty accurate information and signposting to the organisations that can offer support available, there was no other intervention content linked to the theoretical domains to increase the ability of an individual to perform help-seeking behaviour. Rickwood and colleagues (Rickwood et al., 2005) suggested that the belief in one's ability to manage difficulties is important to engage in the help-seeking process. Those with higher self-efficacy are often more likely to recognise the need for help and feel more confident in reaching out (Corrigan et al., 2014). Incorporating intervention content to increase the capability of recipients to seek further help is crucial. For example, rather than just provision of contacts to seek professional help (i.e. the G in the ALGEE approach) to also include how to do this in the session (i.e. via the BCT ‘Demonstration of the behaviour’). While ALGEE is presented as a structured action plan and working through this may be anticipated to contribute to processes such as self-regulation or decision making, there were no BCTs identified that relate to these processes directly. For example, for of the behavioural regulation mechanism of action to be identified, the BCT ‘self-monitoring of behaviour’ would be expected.

Social influence (via encouragement to access to social support) was the only theoretical domain highlighted in the current study that relates to the opportunity component of the COM-B model. However, the decision to seek help for mental health concerns beyond the MHFA sessions has been found to be shaped by the ‘environmental context and resources’ domain. Tóth et al. systematic review (Tóth et al., 2023) found that supportive workplaces characterised by open communication and reduced stigma encourage help-seeking for mental health concerns, especially when employees are made aware of the social and organisational consequences of unaddressed distress. In contrast, high-stress, unsupportive environments can perpetuate stigma, not only discouraging individuals from seeking help but also negatively impacting team cohesion and overall workplace morale (Dewa et al., 2014). Implementation of the ‘information about social and environmental consequences’ BCT defined as ‘providing information about how performing or not performing a behaviour can affect other people or the environment’ may strengthen the ‘E’ (encourage appropriate professional help) component of the MHFA action plan. In a workplace context, an MHFAer could use this technique by highlighting the organisation's commitment to mental health support and emphasising that seeking help is not only accepted but positively contributes to a healthier team dynamic, improved employee wellbeing, and a supportive workplace culture. This reinforces that help-seeking benefits both the individual and the wider work environment.

In addition to capability and opportunity, targeting motivation domains within the COM-B model can further strengthen MHFA interventions in the workplace. One such domain is social and professional role identity, which involves aligning behaviours such as help-seeking with individuals' valued roles. MHFAers can support this by suggesting that recipients view seeking support as consistent with being a responsible colleague, team member, or leader (i.e. framing/reframing BCT). Siebert and Siebert (Siebert & Siebert, 2007) studied over 700 social workers and found that those with a stronger caregiver role identity were less likely to seek help, perceiving it as inconsistent with their professional identity. In workplace contexts, employees who strongly identify with roles that emphasise resilience or caregiving may similarly avoid seeking support. MHFA interventions that frame help-seeking as an aspect of being a responsible and supportive colleague could help to overcome this barrier and enhance overall effectiveness.

Incorporating the BCT ‘identification of self as a role model’, defined as framing the individual's behaviour as an example to inspire others, can enhance both the ‘encourage appropriate professional help’ and ‘encourage other supports’ elements of the ALGEE action plan. For instance, MHFAers can encourage recipients to view themselves as relatable role models who took a positive step by seeking help after an MHFA interaction. This can reduce stigma and promote openness across the workplace.

One methodological limitation of this study is its retrospective approach to identifying the active ingredients and mechanisms of action in the MHFA intervention. This method may naturally limit the depth and accuracy of the data gathered, especially with respect to interview responses, as it relies on recalling and interpreting past events. In this study, we examined data from five first aiders who were interviewed as part of the larger trial and received MHFA support. As such, their reflections may have been influenced by memory bias or shaped by subsequent experiences, potentially diluting the immediacy and contextual richness of their accounts because they were interviewed 6 months after their MHFA training and received their MHFA support at that time. The interview sample was small and purposive, focusing on participants with direct MHFA experience, which restricts generalisability.

A theoretical consideration is the extent to which the BCTs identified are representative of all four MHFA contexts presented in the manual. The training manual chapter and role-play video data sources were limited to the application of the ALGEE approach to psychosis. However, we also examined the other three chapters and found no additional BCTs contained. Six additional BCTs were identified from the delivery of MHFA in practice as demonstrated in the role-play video. Whilst it is possible that these additional BCTs may be biased to the psychosis context, there was some overlap with two of these BCTs also present in the interviews where the mental health contexts did not include psychosis. BCTs were also observed in the training manual that were not present in either the role-play video or the interview accounts. This highlights importance of using triangulation of multiple data sources to identify active ingredients of the intervention. These findings together suggest considerable adaptive work is involved in applying ALGEE in practice. This variability in the application of MHFA may in part explain the little to no effect found on mental health usage in the recent Cochrane review (16) that examined MHFA compared to no intervention. From an implementation standpoint, this emphasises the need to discuss how and when specific BCTs should be operationalised across different settings. Future enhancements to MHFA training might include additional practical examples in the training manual of BCTs to boost their visibility and adoption, thereby enhancing fidelity.

To make the MHFA intervention more effective, it is suggested that future research consider other BCTs and theoretical domains aimed at promoting actual help-seeking behaviours for mental health concerns through a Delphi study and using experimental or quasi-experimental designs to test their impact on help-seeking behaviour. This approach allows for a systematic and theory-informed identification of the most relevant BCTs, refined through expert consensus. Furthermore, future studies can apply the COM-B model and the TDF within qualitative methodologies to identify the individual and environmental changes needed to support help-seeking behaviour in the workplace. This approach can help ensure that MHFA interventions are more effectively aligned with the factors that influence behaviour, increasing the likelihood of achieving their intended outcomes.

## Conclusion

This study offers original and significant insights into the retrospective review of the intervention content and mechanisms of action of MHFA as a mental health literacy intervention. The primary active ingredient of MHFA appears to be social support. Several other behavioural change techniques (BCTs) and intervention functions were identified to encourage mental health-seeking behaviour. However, the MHFA ALGEE action plan could benefit from integrating other BCTs, domains for behavioural change and intervention functions that map onto the COM-B model to enhance its effectiveness in promoting better help-seeking behaviour. Future research should assess the impact of integrating additional BCTs and intervention functions into the MHFA ALGEE action plan to determine the most effective combination for promoting help-seeking behaviour and improving mental health outcomes.

## Supplementary Material

Mental_Health_First_Aiders_Recipients_Interview_questionsCleanVersion.docx Mental_Health_First_Aiders_Recipients_Interview_questionsCleanVersion.docx

## Data Availability

The data supporting this study's findings are available from the corresponding author [OA], upon reasonable request. For the purpose of open access, the author has applied a Creative Commons Attribution (CC BY) licence to any Author Accepted Manuscript version arising.
