# Supplementary material for: Theoretical evaluation of mental health first aid using the behavioural change wheel (BCW)
Source: Health Psychol Behav Med. 2026 Jan 30;14(1):2623324. doi: 10.1080/21642850.2026.2623324 (PMC12862838; doi:10.1080/21642850.2026.2623324)
Supplement: Mental_Health_First_Aiders_Recipients_Interview_questionsCleanVersion.docx [file RHPB_A_2623324_SM3979.docx]

**Mental Health First Aiders**

| **S/N** | **Questions** | **Prompts** |
| --- | --- | --- |
| **1** | **What made you sign up to the MHFA training? (askFocus them to describe their motives in detail) What was the experience of participating in the Online MHFA training?**  How has the experience shaped your understanding of mental health crisis in general? | - Explore their previous knowledge and experience of mental health. - Encourage them to share key learnings from the training. - Ask them to give examples of ways they have support someone following the training. - Explore any further areas they feel the training could have addressed but did not. - Explore how issues around mental health were handled at work before attending the training. Try to understand that this might not relate to them directly so ask them to share any experience that might come to mind. - Focus on how they applied the ALGEE ‘model’, ask for examples of how they have gone through each stage of the model: Approach the person, assist with crises; Listen non-judgmentally; Give support and information; Encourage the person to seek professional help and Encourage them to seek other forms of support. |
| **2** | **How has settling back into work been after the training? How have you found your new role? Has it changed anything? (in terms of acceptance, implementation, and visibility?)** | **Implementation**   - Explore how they have implemented their learning. - Explore practical measures taken to implement learning after training. - Explore the support mechanisms that were in place to support the implementation of learning.   **Visibility**   - Explore what they have done to ensure visibility? (Note that this might not be within their jurisdictions, so you can ask what the organization has done instead). - Explore the support from the organization/senior management to ensure visibility. - Explore the different working situation and how it has affected ensuring visibility.   **Acceptance**   - Explore issues around the acceptance of this form of support by their colleagues. |
| **3** | **Let us talk about instances where you have applied your MHFA skills. Could you provide any examples of when you were able to help someone?** | - Explore any issues around hierarchy that made sessions difficult. - Encourage them to give examples of any difficulty experienced. - Explore how helpful the sessions with recipients has been. |
| **4** | **Has anything changed for you since you became an MHFA? For example, do you feel differently about your relationships with your colleagues, or about the general work culture?** | - Explore what the atmosphere was like beforehand. - Explore what has contributed to the positive or negative atmosphere in the workplace. - Explore any form of stresses associated with playing their role as a MHF-Aider. - Explore with them changes attitudes within the organization, amongst staff members, across hierarchy (Encourage them to give examples) |
| **5** | **Before we roundup, let’s talk about *presenteeism*. What is your understanding of presenteeism? That is people being at work despite not feeling their best or not feeling able to work.**  **(Encourage them to give examples of instances where they have attended work whilst feeling unwell)** | - Explore if their state of being unwell was mental health or physical health related. - Explore the reasons for carrying on with work. - Explore how they feel about carrying on with work despite being unwell. - Explore the potential contributory factors to carrying on with work. - Explore how the difficulty experienced when trying to be open impact on carrying on with work. |
| **6** | **Is there anything you were expecting to be discussed we didn’t talk about? (Use this opportunity to touch on the questions that were not properly explored)** |  |

**Recipients Interview Questions**

| **S/N** | **Questions** | **Prompts** |
| --- | --- | --- |
|  | **Can you tell me a little about the background to the issues that you shared with the mental health first aider?** | - When did they first arise? - How did they develop over time? - What led to their decision to share at work? - What led to the decision to the approach MHFA more specifically? |
|  | **After you made the decision to approach the MHFA, what was your experience of sharing your concerns at work?** | - Explore what sort of experiences they have shared with someone at work. - Ask them who they shared their experiences with? i.e. did they share with a colleague and then an MHFA or did they go straight to the MHFA? - Explore how they felt about these experiences and how they managed their feelings. - Explore the barriers to sharing their concerns/experiences at work. |
|  | **Following the concerns discussed earlier, do you consider these issues to be linked to your mental health? If so, what is your understanding of how they are linked?** | - Explore their understanding of mental health. - Explore whether they think the mental health issues are due to long standing issues and/or life-events or are more specifically related to work stresses. - Do they think their ‘well-being’ is compromised by work issues? - How do they think about mental health issues? As an illness or a response to events that have occurred…? Or both…? - Were there any barriers in being able to talk about any of these concerns? |
|  | **After you had spoken to the MHFA, how would you describe the kind of support you received and the ways in which it affected your feelings and your mental health?**  **(Note to researcher: With regards to daily work? Community? Family?)**  **(Encourage them to give examples)** | - Explore the support available both at work & outside work. - Ask them to describe what specific qualities the MHFA was able to deliver – this should be quite detailed, so ask for examples and particular incidences of positive and/or negative. - Ask them about any other forms of support they received (in addition to the MHFA). (At work or Outside work) (Formal or Informal) - Explore the impact of the pandemic on the sort of support that was available. (At work or outside work) - Find out how helpful the support they explored. (Encourage them to give examples). - Explore the impact of the pandemic on how they manage their feelings. |
|  | **Let us talk about your encounter with your MHF-Aider, what are your thoughts on the help you received?**  **How did the opportunity come about?**  **What do you think is the purpose of MHFA?** | - Explore how helpful MHFA was. - Explore how they felt about their encounter with the MHF-Aider. (Safety, warmth, confidentiality) - Explore the impact of the help received from the MHF-Aider on their concerns raised earlier. - Explore further the impact on relationships at work? |
|  | **Before we roundup, let’s talk about *presenteeism*. What is your understanding of presenteeism? That is people being at work despite not feeling their best or not feeling able to work.**  **(Encourage them to give examples of instances where they have attended work whilst feeling unwell)** | - Explore if their state of being unwell was mental health or physical health related. - Explore the reasons for carrying on with work. - Explore how they feel about carrying on with work despite being unwell. - Explore the potential contributory factors to carrying on with work. - Explore how the difficulty experienced when trying to be open impact on carrying on with work. - In your view, is there any benefit from presenteeism (to the individual to the organisation)? |
|  | **Is there anything you were expecting to discuss that we didn’t talk about? (Use this opportunity to touch on the questions that were not properly explored)** |  |
